# Clinical, Serological, and Molecular Observations from a Case Series Study during the Asian Lineage Zika Virus Outbreak in Grenada during 2016

**DOI:** 10.1155/2018/4635647

**Published:** 2018-02-01

**Authors:** Marco Brenciaglia, Trevor P. Noël, Paul J. Fields, Satesh Bidaisee, Todd E. Myers, William M. Nelson, Neeraja Venkateswaran, Kodumudi Venkateswaran, Nishanth Parameswaran, Avi Bahadoor, Katherine Yearwood, Veronica Mapp-Alexander, George Mitchell, A. Desiree LaBeaud, Calum N. L. Macpherson

**Affiliations:** ^1^St. George's University School of Medicine, True Blue Campus, St. George's, Grenada; ^2^Windward Islands Research and Education Foundation, St. George's, Grenada; ^3^Naval Medical Research Center, Silver Springs, MD 20910, USA; ^4^Tetracore, Inc., Rockville, MD 20850, USA; ^5^Omni Array Biotechnology, LLC, Rockville, MD 20850, USA; ^6^Ministry of Health, Ministerial Complex, St. George's, Grenada; ^7^Stanford University, Stanford, CA 94305, USA

## Abstract

This paper describes the spatial and temporal distribution of cases, demographic characteristics of patients, and clinical manifestations of Zika virus (ZIKV) during the 2016 outbreak in Grenada. The first reported case was recorded in St. Andrew Parish in April, and the last reported case was seen in November, with peak transmission occurring in the last week of June, based on test results. Data were collected from a total of 514 patients, of whom 207 (40%) tested positive for ZIKV. No evidence was found that testing positive for ZIKV infection was related to age, gender, or pregnancy status. Clinical presentation with rash (OR = 2.4, 95% CI = 1.5 to 3.7) or with lymphadenopathy (OR = 1.7, 95% CI = 1.0 to 2.9) were the only reported symptoms consistent with testing positive for ZIKV infection. During the Zika outbreak, the infection rate was 20 clinical cases per 10,000 in the population compared to 41 cases per 10,000 during the chikungunya outbreak in Grenada in 2014 and 17 cases per 10,000 during the dengue outbreak in 2001-2002. Even though the country has employed vector control programs, with no apparent decrease in infection rates, it appears that new abatement approaches are needed to minimize morbidity in future arbovirus outbreaks.

## 1. Introduction

Seventeen percent of all disease cases worldwide, or more than one billion occurrences annually, are from vector-borne diseases, resulting in over one million deaths. The vectors responsible for transmitting these diseases include mosquitoes, ticks, flies, and even certain aquatic snails. With increasing trade, international travel, and average global temperatures, vector-borne outbreaks could increase in severity and frequency [1–3]. Thus, it is increasingly important that these diseases are documented, surveyed, and studied to mitigate the morbidity and mortality resulting from their infections. Although much research has focused on studying these diseases, there is limited information regarding their occurrence and persistence in small island nations such as Grenada.

Two mosquito species, *Aedes aegypti* and *Aedes albopictus*, are efficient transmission vectors for the yellow fever, dengue (DENV), chikungunya (CHIKV), and Zika (ZIKV) viruses [4–9]. Arthropod-borne flaviviruses can cocirculate and are known to cause coinfections [5–12]. The vectors thrive primarily in tropical and subtropical areas, but have been spreading into temperate climates as well [3]. Currently, 174 countries and territories, including Grenada, host stable populations [13].

In Grenada, *Aedes aegypti* was responsible for the transmission of dengue fever during late 2001 and early 2002 and chikungunya fever in 2014 [14, 15]. Although originally from Central Africa where the ancestral *Aedes aegypti* formosus bred in riverbeds, tree holes, and rock pools, *Aedes aegypti* today breeds in clean domestic and peridomestic water sources and exhibits both domestic and sylvatic life cycles [16–19].

## 2. Materials and Methods

An outbreak of Zika fever occurred in Grenada from late April to early November 2016. In partnership with the Grenada Ministry of Health, the Zika Research Team at the Windward Islands Research and Education Foundation (WINDREF) provided patient survey forms to healthcare facilities throughout the country for clinicians to record and to report patient demographic and symptomatic information. The forms were designed to collect information on 20 symptoms that were considered for surveillance purposes as indicative of possible ZIKV infection. Patient enrollment in this study was optional and contingent upon informed consent. Prior to enrollment, all participants were given counseling about the aims of the study and the potential risks of participation. Ethical approval of this study was given by the St. George's University Institutional Review Board.

Physicians provided the WINDREF Zika Research Team with whole blood and urine samples collected from Grenadian residents who voluntarily went to healthcare facilities located across the tri-island nation of Grenada during the outbreak. Samples were drawn daily from all patients suspected with possible ZIKV infection. Testing of the samples was provided by WINDREF as a public service to the community.

The laboratory at the Grenada General Hospital spun the blood samples to separate the sera, aliquoted each sample into two subsamples, and sent one to the Caribbean Public Health Agency in Trinidad for testing and the other to WINDREF. At WINDREF, the samples were further aliquoted into two subsamples. One of the subsamples was tested at Tetracore, Inc., and Omni Array Biotechnology, LLC, both headquartered in the United States. The other subsample was stored at WINDREF for archival purposes.

A total of 626 clinical samples were collected from 514 total patients. The clinical samples comprised 523 sera, 102 urine, and one cerebrospinal fluid (CSF). The 523 serum samples were collected from 480 patients as some samples were repeat samples collected at different times.

The diagnostic assays used in the study had been developed by Tetracore and Omni Array Biotechnology. The serum samples were tested with real-time reverse transcription polymerase chain reaction (rRT-PCR) and multiplex magnetic bead-based immunoassay to detect IgM and IgG responses to ZIKV, DENV, CHIKV, and other related flaviviruses [20]. The urine samples were tested with rRT-PCR only. The multiplex rRT-PCR assay was designed to detect viral RNA for ZIKV, DENV, and CHIKV and includes an internal control to ensure the validity of the test. The primer and probe selections were confirmed by in silico analysis, and then, the assay was optimized using spiked samples. The test was optimized using both extraction of RNA and direct testing of the samples. The testing was performed in a 96-well plate format in a laboratory-based platform. The multiplex magnetic bead-based immune assay was designed to measure human IgM to the closely related arboviruses ZIKV, DENV (serotypes 1, 2, 3, and 4), CHIKV, West Nile, yellow fever, Japanese encephalitis, and tick-borne encephalitis. The test also included five different internal controls that ensure the quality of the assay and are used for normalization of the results. Patient samples that were positive by rRT-PCR were considered *confirmed* cases, and those positive only by IgM serology were considered *presumptive* recent infections. Further details about the tests are described in the appendix.

In areas like Grenada where several flaviviruses cocirculate, nonspecific flavivirus responses can occur due to cross reactivity between immunoglobulins against closely related viruses [21, 22]. Plaque reduction neutralization tests (PRNTs) are highly specific and thus are useful for identifying antibodies against closely related virus species and are considered the gold standard for diagnosis [7, 23]. PRNT was not performed due to the complex, time-intensive, and labor-intensive nature of the assay. There were two patients that were positive by rRT-PCR for CHIKV, one of whom tested positive for anti-ZIKV IgM. Although these patients were possibly infected with ZIKV, they were excluded from the study results to be conservative since definitive diagnosis was not possible.

A patient was deemed *symptomatic* if the attending physician recorded for that patient at least one of the symptoms considered to be possible indicators of ZIKV infection. For symptomatic patients whose blood samples tested positive for ZIKV by rRT-PCR, the number of days between reported symptom onset and sample collection ranged from 0 to 8 days with a median of 3 days. For symptomatic patients whose blood samples tested positive for IgM against ZIKV, the number of days between reported symptom onset and sample collection ranged from 0 to 31 days with a median of 4 days. For symptomatic patients whose urine samples tested positive for ZIKV by rRT-PCR, the number of days between reported symptom onset and sample collection ranged from 2 to 9 days with a median of 3.5 days.

## 3. Results

### 3.1. Spatial and Temporal Distribution of Cases

Clinical samples were collected from 514 total patients, of which 207 (40%) tested positive by ZIKV rRT-PCR or ZIKV IgM or both. Nine samples tested positive by both tests. Testing sera from 480 patients resulted in 107 positives for ZIKV by rRT-PCR and 109 positives for ZIKV IgM. Of the 102 urine samples, 12 tested positive for ZIKV by rRT-PCR. The one CSF sample tested negative by rRT-PCR.

Table 1 summarizes the spatial and temporal occurrences of reported cases, the number of symptomatic and asymptomatic cases, the number of positive and negative cases for each parish and for the country. Also shown is the number of Guillain-Barré syndrome (GBS) cases identified by the Grenada Ministry of Health. The parishes on the main island are listed in decreasing order of population size with the neighboring island of Carriacou listed last. The parish population data are based on the 2011 Grenada Census data [24].

The outbreak began in the last week of April in St. Andrew Parish when a 28-year-old female tested positive for ZIKV by rRT-PCR. Her reported symptoms included rash, fever, headache, joint pain, body pain, lymphadenopathy, nausea or vomiting, and diarrhea. This patient is considered the index case for the country [25].

To make relative comparisons among geographic areas across the country, an *indicated attack rate* was calculated for each parish as the proportion of positive samples collected from a parish scaled to the size of the parish. With a population of 25,722 people and 36 positive cases, St. Andrew Parish had an indicated attack rate of 14 cases of ZIKV infection per 10,000 people. Of the 36 positive cases, 18 were rRT-PCR positive and 18 were ZIKV IgM positive. The positive cases collected in St. Andrew Parish were 17% of the total ZIKV-positive cases identified in this study.

St. Mark Parish, in the northwest of the country, was the next to report its first case, during the week of May 8. St. Mark Parish has a population of 4,086 with 12 positive cases reported, of which eight tested rRT-PCR positive and four tested ZIKV IgM positive, giving an indicated attack rate of 29 positive cases per 10,000 in the population. St. Mark Parish had 6% of the ZIKV-positive cases identified in this study.

St. George Parish, in the southwest, with a population of 36,823, was the next to report its first case in late May. With 117 positive cases of which 66 were rRT-PCR positive and 51 were ZIKV IgM positive, St. George Parish had an indicated attack rate of 32 per 10,000 people in the population. St. George Parish had 57% of the ZIKV-positive cases identified in this study. With the highest population density in the country, St. George Parish was also the focus of infection during the outbreaks of DENV in 2001-2002 and CHIKV in 2014.

After St. George Parish, three other parishes reported their first cases within two weeks of each other: St. Patrick Parish in the week of June 5, St. David Parish in the week of June 12, and St. John Parish in the week of June 26. These three parishes combined had 16% of the total positive cases identified in this study.

Carriacou, located northeast off the coast of St. Patrick Parish, and accessible only by a ferry or plane, was the last parish to have its first diagnosed case of ZIKV, which was reported during the week of July 3. Carriacou had only 4% of the total positive cases identified in this study.

The countrywide-indicated attack rate during the outbreak was 20 per 10,000 people with peak transmission occurring in the week of June 26 with 26 cases, of which 21 were rRT-PCR positive and 5 were ZIKV IgM positive. The reported date of symptom onset was used as the week of indicated infection. When the symptom onset date was not available, the date of sample collection was used as a conservative infection date.

**Figure 1:**
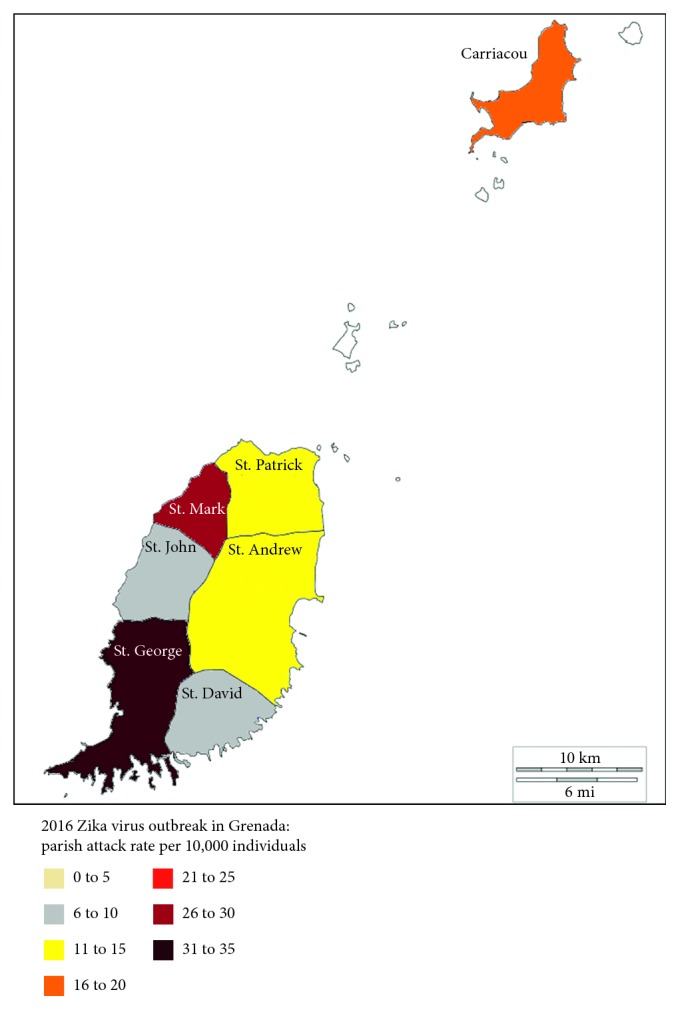
Spatial distribution of ZIKV-positive cases by parish during the 2016 Zika outbreak in Grenada showing the majority of cases concentrated in the urban area of the country.

Figure 1 shows the spatial distribution of all ZIKV-positive cases with the highest concentration in St. George Parish in the southernmost area, which is the most urban area.

**Figure 2:**
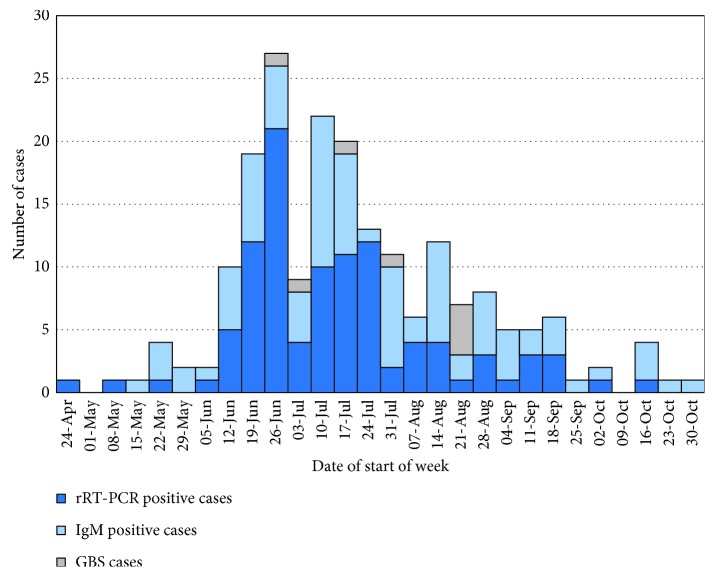
Temporal distribution of positive cases by week during the 2016 Zika outbreak in Grenada showing the outbreak that lasted for 28 weeks with the majority of cases concentrated between the weeks of June 12 and September 18. The Guillain-Barré cases occurred between June 26 and August 21.

Figure 2 presents graphically the temporal distribution of ZIKV-positive cases during the outbreak by week. The rRT-PCR-positive cases are shown in dark blue and the ZIKV IgM-positive cases are shown in light blue with the GBS-positive cases identified by the Grenada Ministry of Health shown in green.

### 3.2. Demographic Characteristics of Patients

The age distribution of the sample ranged from one-day old to 90 years old, with a median age of 30 years. The age distribution for positive cases ranged from one year old to 90 years old with a median age of 31. Of 73 patients under the age of 20, 28 (38%) tested positive for ZIKV, of whom 7 were rRT-PCR positive and 21 were ZIKV IgM positive, while of the 433 patients of 20 years of age and older, 177 (41%) tested positive, of which 107 were rRT-PCR positive and 70 were ZIKV IgM positive. Eight patients did not specify their ages. There was no evidence that the rate of infection was different between the age groups (test of proportions, *p* > 0.05).

Of the 131 males in the study, 58 (44%) tested positive for ZIKV with 30 rRT-PCR positives and 28 ZIKV IgM positives. Of the 380 females in the study, 148 (39%) tested positive for ZIKV with 85 rRT-PCR positives and 63 ZIKV IgM positives. Three patients did not specify their gender. There was no evidence that the rate of infection was different between genders (test of proportions, *p* > 0.05).

Of the 380 female patients in the study, 117 (31%) were pregnant, while 260 female patients were not pregnant. The pregnancy status was unknown for three female patients. Of the pregnant patients, 45 (38%) tested positive for ZIKV, of which 28 tested as rRT-PCR positive and 17 tested as ZIKV IgM positive, while 99 (38%) of the nonpregnant patients tested positive for ZIKV, of which 53 tested as rRT-PCR positive and 46 tested as ZIKV IgM positive. Consequently, there was no evidence indicating that the rate of infection was related to pregnancy status (test of proportions, *p* > 0.05). Of the 45 women that were pregnant and who tested positive for ZIKV infection, the trimester of their pregnancy was known for 17 with six in the first trimester, five in the second trimester, and six in the third trimester.

### 3.3. Clinical Manifestations of the Zika Virus

Of the 514 patients enrolled in this study, 424 (82%) were symptomatic with 191 (45%) testing positive, of which 107 were rRT-PCR positive and 84 were ZIKV IgM positive, and 233 (55%) testing negative for ZIKV.

Among the 424 symptomatic cases, 191 tested positive, while 233 tested negative. Among the 90 asymptomatic cases, 82 (91%) were women, all of whom were undergoing antenatal screening. Of the eight asymptomatic cases (all males), only one tested positive for ZIKV. Furthermore, 90 of 514 (18%) patients were asymptomatic with 16 (18%) testing positive, of which 8 were rRT-PCR positive and 8 were ZIKV IgM positive, and 74 (82%) testing negative for ZIKV.

Table 2 shows the ten most common symptoms reported among symptomatic cases. The symptoms are listed in descending order of each symptom's indicated distinctiveness between positive and negative cases. Although a broad range of symptoms were reported in the symptomatic patients, most symptoms occurred so infrequently that they did not provide any information that could be considered as indicative of ZIKV. The odds ratio (OR) for a symptom being indicative of a patient testing positive versus negative is shown in the rightmost column of Table 2. Symptomatic patients were more likely to test positive than asymptomatic patients (OR = 3.8, 95% confidence interval 2.1 to 6.7, *p* < 0.001).

Of the ten symptoms listed in Table 2, rash was the most indicative of distinguishing between positive and negative cases (OR = 2.4, *p* < 0.001). The only other symptom that was a possible distinctive indicator of ZIKV was lymphadenopathy (OR = 1.7, *p* < 0.05).

Guillain-Barré syndrome, which presents with severe and sometimes life-threatening transient paralysis, has been noted to occur during Zika outbreaks around the world [26–32]. There were nine cases of GBS during the 2016 Zika outbreak in Grenada; however, only eight cases were enrolled in this study. The earliest case of GBS was reported during the week of June 26, the week of peak ZIKV transmission, while the last cases was reported during the week of August 21. Four GBS cases were male and five were female. The affected patients ranged in the age from 21 to 67 years old, with a median of 41 years.

Of the eight GBS cases enrolled in this study, four tested positive by IgM serology for ZIKV, two had nonspecific anti-flavivirus IgM, and two patients showed no evidence of ZIKV infection, though one of these negative cases was lost to serologic follow-up. Six cases of GBS were tested by IgG ELISA and all six cases tested positive for ZIKV, while five also tested positive for DENV and four tested positive for CHIKV. There was evidence that as the number of ZIKV-positive cases increased across the parishes, the number of GBS cases tended to increase as well (*r* = 0.89, *p*=0.007).

## 4. Discussion

### 4.1. Spatial and Temporal Distribution of Cases

The ZIKV vector, *A. aegypti*, is known to be an endophagic urban dweller that clusters around population centers [33–36]. In past outbreaks in Grenada of other arboviruses carried by the same mosquito species, specifically the DENV (2001-2002) and CHIKV (2014) outbreaks, cases tended to cluster in population centers [14, 15]. This was observed again during the ZIKV (2016) outbreak.

St. George Parish, which is the most densely populated parish with 36% of Grenada's population, had 57% of the positive cases identified in this study. St. Andrew Parish with 25% of the population had 17% of the reported positive cases. St. Mark Parish and St. George Parish, despite having, respectively, the lowest and highest populations, had the highest indicated attack rates. It is reasonable to conjecture that this could be because these parishes had early cases of the disease, and thus, there was ample time for the virus to circulate within their immunologically naïve populations.

### 4.2. Demographic Characteristics of Patients

Although testing positive for ZIKV infection was not found to be related to age group, gender, or pregnancy, of the 117 pregnant women enrolled in the study, 67 (57%) were asymptomatic, compared to an asymptomatic rate of 5% (12 of 260) among nonpregnant women (test of proportions, *p* < 0.001). It is possible that foreknowledge of the effects of ZIKV on a maturing fetus and fearing the potential negative health effects on their unborn fetuses motivated pregnant women to enroll in the study to obtain a screening test even though they did not display symptoms, while nonpregnant women enrolled in the study for the screening test due to being symptomatic.

Of the 45 pregnant women who tested positive for ZIKV, 11 were in their first or second trimester of pregnancy. The first and second trimesters are thought to bear the highest risk of congenital Zika virus syndrome in patients with symptomatic ZIKV infection. As is well known, this syndrome can have a range of negative health consequences for a newborn, and thus, these cases are of particular concern [31, 37–40]. Although the incidence rate of microcephaly has been estimated generally to affect 1–4% of newborns of mothers who are symptomatic and test positive for ZIKV infection during their first trimesters, the effects of ZIKV infection in asymptomatic cases are less well understood [38, 41–44]. Occurrences of microcephaly in Grenada and the potential for congenital and later effects in children of ZIKV-positive mothers are topics of continuing study by the WINDREF Zika Research Team.

### 4.3. Clinical Manifestations of the Zika Virus

Consistent with the symptoms reported in Zika outbreaks in other countries, the most commonly observed symptoms in cases that tested positive for ZIKV in Grenada were similar to the symptoms of the two other potentially cocirculating flaviviruses CHIKV and DENV. The similarity of these symptoms means that an accurate diagnosis of ZIKV infection can be challenging and requires both knowledge of symptoms that predict infection and laboratory testing of blood and urine samples.

Laboratory diagnostic methods for viral infection include detection of specific virus in the test sample, molecular methods such as rRT-PCR which provides evidence of the viral genomic material in an infected sample, and immunodiagnostic methods, which provide evidence of antigens or antibodies to the virus. Detection of virus or the viral genomic material is possible in the acute phase of the disease. Serological methods may be used for determination of primary or secondary infections, depending on the immunological status of an infected patient. Primary response is seen in a patient who has not been exposed to a viral infection, while a secondary response is seen in a patient who may have had a past infection. Detection of antibodies in acute- and convalescent-phase sera is traditionally made by IgM or IgG ELISAs. Traditional serological methods, such as the plaque reduction neutralization test (PRNT), are complex and are both time- and labor-intensive. IgM-capture ELISA (MAC-ELISA) tests are also time-consuming, taking up to two days from sample to result.

Serological diagnosis of a flavivirus infection, such as ZIKV or DENV fever, is also complicated by the fact that in endemic areas, patients may have multiple and sequential infections with different flaviviruses [18]. It has been shown in the past that most patients with primary DENV infections may not show detectable DENV IgM or IgG after four months of infections, but patients with secondary infections may show detectable levels for a much longer period [45].

Based on the results in this study, the symptoms that can be considered predictive of a patient testing positive for ZIKV were rash (OR = 2.4, 95% CI = 1.5 to 3.7) and lymphadenopathy (OR = 1.7, 95% CI = 1.0 to 2.9). The presence or absence of these symptoms can help clinicians in Grenada diagnose future ZIKV infections. Notably, although conjunctivitis has been observed to be a differential diagnostic symptom for ZIKV infection in other studies in other countries, there was no evidence that it was a predictive symptom during the outbreak in Grenada [7, 44, 46].

Since the patients enrolled in this study were patients who had voluntarily gone to a medical facility seeking treatment, 92% of the positive cases in this study were symptomatic compared to the typically observed symptomatic proportion of only 20–25% of ZIKV-infected cases. Correspondingly, 76% of the negative cases in this study were symptomatic, indicating perhaps a tendency for people to seek testing when they have symptoms during a known Zika outbreak.

Consequently, the patients enrolled in this study consisted largely of symptomatic individuals (82%), but the symptomatic rate in the general population was likely to have been lower. If the symptomatic rate of infected individuals in Grenada was closer to the typical rate and if the rate of infection was the same in the general population among people who did not seek medical care for ZIKV infection during the outbreak, then an estimated total of about 18,400 people were infected during the outbreak with an estimated attack rate in the general population of 17.8% (17.7% to 18.0%).

Of the nine cases of GBS that occurred during the Zika outbreak, the date of onset was known for eight cases. The dates of onset occurred during an eight-week period and coincided both spatially and temporally with high rates of ZIKV infection. With a global annual incidence of GBS estimated between 1 to 2 per 100,000 individuals, if all nine cases of GBS that occurred during this eight-week period were to continue year round, this would represent 30 to 50 times the worldwide average annual incidence rate [30, 47, 48]. This dramatic increase in incidence and the strong correlation between the number of Zika and GBS cases in each parish, combined with the fact that six of the eight cases of GBS in this study displayed evidence of recent flavivirus infection, add to the growing body of evidence of higher rates of GBS coinciding with Zika outbreaks and suggesting a possible link between ZIKV infection and the onset of GBS [7, 26–32].

## 5. Conclusions

This paper has described the spatial and temporal distribution of cases, demographic characteristics of patients, and clinical manifestations of cases of the presumed Asian lineage of ZIKV transmitted by *Aedes aegypti* in the small Caribbean island nation of Grenada in 2016. The outbreak lasted for 28 weeks with infections reported in all areas of the country and concentrated in urban areas. Only rash and lymphadenopathy were consistent with testing positive for ZIKV infection, with rash being the most distinguishingly indicative. Definitive syndromic diagnosis alone was not possible due to symptoms overlapping with potentially cocirculating CHIKV and DENV, thus making laboratory confirmation essential.

During the Zika outbreak, the infection rate was 20 clinical cases per 10,000 in the population. In comparison, the infection rate of clinical cases of CHIKV in Grenada in 2014 was 41 cases per 10,000, while it was 17 per 10,000 for DENV in 2001-2002 [14, 15]. Even though common vector control methods, such as antimosquito fogging and breeding source reduction, have been implemented in Grenada and, in fact, intensified during arbovirus outbreaks, it appears that they have had minimal effect on reducing the impact of mosquito-borne viruses, indicating perhaps that new and novel approaches to vector control are needed to prevent future arbovirus outbreaks.

## Figures and Tables

**Figure 3:**
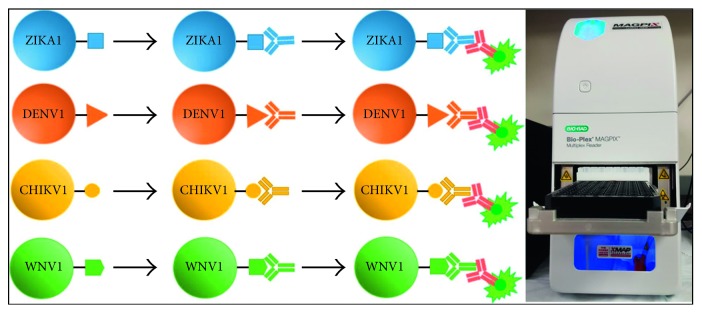
Illustration of the principle of a multiplex serodiagnostic assay used in the study.

**Table 1:** Summary of the spatial and temporal distribution of cases by parish during the 2016 Zika outbreak in Grenada showing the first reported case in St. Andrew Parish in the week of April 24 and the last reported case in St. George Parish in the week of October 30.

Parish	Population	Week of first case reported	Week of maximum cases reported	Week of last case reported	Symptomatic cases (positive, negative)	Asymptomatic cases (positive, negative)	GBS cases
St. George	36,823	May 22	June 26	October 30	110, 132	7, 38	4
St. Andrew	25,722	April 24	July 17	September 11	35, 48	1, 19	2
St. David	12,561	June 12	June 26, July 17, and August 14	August 28	12, 23	0, 5	0
St. Patrick	10,980	June 5	July 31	October 16	13, 17	2, 3	1
St. John	7,802	June 26	July 10	August 28	6, 6	0, 1	0
St. Mark	4,086	May 8	June 26	October 2	10, 5	2, 3	1
Carriacou	5,354	July 3	July 3	September 4	5, 2	4, 5	1
Grenada	103,328	April 24	June 26	October 30	191, 233	16, 74	9

**Table 2:** Symptoms most commonly reported among symptomatic cases during the 2016 Zika outbreak in Grenada, showing the odds ratio for testing positive or negative with rash as statistically highly significant (^∗∗^*p* < 0.001) and the odds ratio for lymphadenopathy as statistically significant (^∗^*p* < 0.05).

Symptom indicative of testing positive for ZIKV	ZIKV-positive cases (proportion of 191)	ZIKV-negative cases (proportion of 233)	Odds ratio (95% CI)
Rash^∗∗^	154 (81%)	150 (64%)	2.4 (1.5 to 3.7)^∗∗^
Lymphadenopathy^∗^	38 (20%)	30 (13%)	1.7 (1.0 to 2.9)^∗^
Nausea/vomiting	38 (20%)	32 (14%)	1.6 (0.9 to 2.6)
Diarrhea	26 (14%)	28 (12%)	1.2 (0.7 to 2.1)
Fever	112 (59%)	137 (59%)	1.0 (0.7 to 1.5)
Headache	74 (38%)	92 (40%)	1.0 (0.7 to 1.4)
Chills	48 (25%)	58 (25%)	1.0 (0.7 to 1.6)
Eye pain	67 (35%)	86 (37%)	0.9 (0.6 to 1.4)
Body pain	68 (36%)	98 (42%)	0.8 (0.5 to 1.1)
Joint pain	97 (51%)	144 (62%)	0.6 (0.4 to 1.0)

**Table 3:** Details of the rRT-PCR assay used in testing for CHIKV, DENV, and ZIKV, including the genome regions targeted, the fluorescent dyes, and the quencher.

	Assay	Target	Fluorescent dye	Quencher
(1)	Chikungunya (CHIKV)	NP 3 region	DFO	BHQ
(2)	Dengue (DENV)	3′ UT conserved region	FAM	BHQ
(3)	Zika (ZIKV)	NS 5 region	Texas red	BHQ
(4)	Internal control (IC)	Synthetic oligo	ATTO 647	BHQ

**Table 4:** rRT-PCR analytical evaluation showing that sensitivity and specificity are effectively 100% for ZIKV.

Test evaluation result	Present	Absent	Subtotal	Total
CHIKV positive	16	1	17	56
CHIKV negative	0	39	39	
DENV positive	16	0	16	56
DENV negative	0	40	40	
ZIKV positive	16	0	16	56
ZIKV negative	0	40	40	
*Analysis*	*CHIKV*	*DENV*	*ZIKV*	
Sensitivity	100.0%	100.0%	100.0%
Specificity	97.5%	100.0%	100.0%
Positive predictive value	94.1%	100.0%	100.0%
Negative predictive value	100.0%	100.0%	100.0%

## Appendix

A description of the laboratory tests used in the study is presented below.  Table 3 presents the details of the multiplex rRT-PCR assay developed by Tetracore, Inc.

The four-plex rRT-PCR assay used includes all reagents to amplify CHIKV, DENV (serotypes 1, 2, 3, and 4), and ZIKV viral RNA and synthetic target as an internal control (IC) to monitor the test performance. The primer and probe sequences are proprietary to the manufacturer. Analytical sensitivity and specificity of the assay were determined using 16 contrived serum samples made with each cell culture-derived virus. Eight uncontrived samples were used as negative controls. Additionally, each of the 16 contrived samples was negative for two other viruses, and a total of 40 negative samples were thus arrived at for each target. DENV assay was designed as PAN assay that targets 3′ UT region and can detect all four serotypes of DENV. CHIKV assay targets the NP3 region, and ZIKV assay targets the NS5 region of viral genomic RNA.  Table 4 shows the analytical evaluation results for the assay along with the sensitivity and specificity calculations.

To detect IgM and IgG responses, various recombinant arboviral antigens were coupled to optically coded microspheres from Luminex Corporation. This fluorescent-labeled microsphere assay was used to differentiate between recent and past arbovirus infections or coinfections that may occur in endemic regions. For ZIKV, two different NS1 antigens and one envelop antigen were used. For other flaviviruses, NS1 antigens were used. For CHIKV, two different envelop antigens were used. Plasma samples from nonhuman primates (NHPs) prior to and after ZIKV infection were used for longitudinal assessment of IgM and IgG antibody responses for evaluation of this method.  Figure 3 depicts the principle of a multiplex serodiagnostic assay that was developed by Tetracore, Inc., and Omni Array Biotechnology, LLC.

The development of the multiplex serodiagnostic test was presented at the American Association of Immunologists annual conference, *Immunology 2017*, 12–16 May 2017, in Washington, DC. The abstract was published in the *Journal of Immunology*, and it can be accessed at http://www.jimmunol.org/content/198/1_Supplement/81.26.
